# Supplementary material for: Estimating COVID-19 mortality in Italy early in the COVID-19 pandemic
Source: Nat Commun. 2021 May 12;12:2729. doi: 10.1038/s41467-021-22944-0 (PMC8115692; doi:10.1038/s41467-021-22944-0)
Supplement: Supplementary file 1 — Supplementary Information [file 41467_2021_22944_MOESM1_ESM.pdf]

1 Supplementary Information : How deadly is COVID-19? A rigorous analysis of excess mortality and age-dependent fatality rates in Italy

Supplementary Methods

We estimate the true mortality count due to COVID-19 by comparing the current mortality to a prediction derived from the historical mortality in different regions of Italy. Specifically, we construct a counterfactual for every region, i.e. the expected mortality count under the scenario that the pandemic had not occurred. For age stratified rates, we also construct a counterfactual for different age groups. It is the best prediction given the historical probability distribution of the death rate time series data, combined with the trend in the 2020 mortality data before the beginning of the pandemic. This approach is superior to the averaging of historical data in that it can account for the trends that may be correlated in time. We then compare this counterfactual with the reported total mortality numbers for 2020 to obtain an excess death rate.

In the language of statistical analysis, we treat the past years 2015-2019 as control units and the current year 2020 as a treated unit. There are $N = 5$ control units of 25-week time-periods from Jan 1st to June 27th ($T = 25$). Since Italy reported its first death due to COVID-19 on February 22nd 2020, a conservative estimate is that the pandemic of COVID-19 started the week of February 16th 2020 with respect to mortality. We refer to this time as $T_0 = 6$. We ignore the changes in population over the past five years: between 2016 and 2020 the Italian population changed by 0.3%, and the relative demographics in terms of age and sex distributions changed by less than 0.5%.

For the ease of mathematical notation, we define some matrices corresponding to the mortality data. Let $Y_0 = [X_0, Z_0]$ and $Y_1 = [X_1, Z_1]$ represent the matrix for the mortality (total number of weekly deaths due to any cause) in control units and treated unit, respectively, in the absence of any pandemic, where X and Z represent the pre- and post-February 16th blocks of the matrix i.e. every row of Y is a different control unit and every column is a new week. Then the shapes of different matrices are - $Y_0 : N \times T$, $Y_1 : 1 \times T$, $X_0 : N \times T_0$, $Z_0 : N \times (T - T_0)$ and correspondingly for X_1 and Z_1. Since the treated unit undergoes a pandemic, the observed mortality is different from Y_1. We refer to it as $Y_1^P = [X_1^P = X_1, Z_1^P]$. Note that before February 16th 2020, Y_1 and Y_1^P are the same. Given the data from the previous years, Y_0, and the current data, Y_1^P, we are interested in predicting the counterfactual Y_1 in the absence of the pandemic. This can then be compared to the observed data Y_1^P to assess the effect of the pandemic after February 16th 2020.

The simplest counterfactual is to model the mortality count in 2020 by the mean of historical data $\overline{Y}_0$,

$$Y_1 = \overline{Y}_0 = \frac{1}{N} \sum_{i=1}^{N=5} Y_{0,i}. \tag{1}$$

This, however, is completely agnostic of the observed pre-pandemic data and ignores time trends that may help improve the counterfactual. We improve on this model with two alternatives, a Conditional Mean with a Gaussian process (CGP), and a Synthetic Control Method (SCM).

CGP for the counterfactual analysis[1] assumes a Gaussian distribution of the data and and estimates its mean and covariance from the historical data. For simplicity, we simply learn the covariance matrix from the historical data. However given the small size of control units (N=5) as compared to the number of weeks (T=25), this can be noisy. To regularize it, we do a principal component analysis of the control units data and find that the dominant 2 principal components explain more than 90% of the variation observed in the control data. Hence we keep only these 2 components and this removes the small time-scale noise from the covariance matrix. This choice provides a good trade-off between capturing the variations in the data while avoiding over-fitting. We assume no noise, which means that by construction the CGP predictions exactly match the data points for pre-pandemic data points. The associated data covariance matrix can be written in block-form is $\Sigma_{YY_0} = \begin{bmatrix} \Sigma_{XX_0} & \Sigma_{XZ_0} \\ \Sigma_{ZX_0} & \Sigma_{ZZ_0} \end{bmatrix}$.

The counterfactual Y_1 follows the same distribution as the control units, i.e. a multivariate Gaussian with mean $\overline{Y}_0 = [\overline{X}_0, \overline{Z}_0]$ and covariance Σ_{YY_0}. We are interested in the prediction of post-pandemic Z_1: the conditional mean given the pre-pandemic data X_1^P and the post-pandemic control mean $\overline{Z}_0$. For this we can simply use the standard formula for conditional mean and covariance of multi-variate Gaussian distribution i.e.

$$Z_1 = \overline{Z}_0 + \Sigma_{ZX_0} \Sigma_{XX_0}^{-1} (X_1^P - \overline{X}_0), \tag{2}$$

and the corresponding covariance matrix is

$$\Sigma_{ZZ_1} = \Sigma_{ZZ_0} - \Sigma_{ZX_0} \Sigma_{XX_0}^{-1} \Sigma_{XZ_0}. \tag{3}$$

We use the diagonals of this covariance matrix as the error on the predicted counterfactual.

SCM[2], our second approach, is a data driven method. It makes minimal assumptions regarding the underlying data distribution. Instead, it makes the assumptions about the causal structure that there is a weighted average of the control units

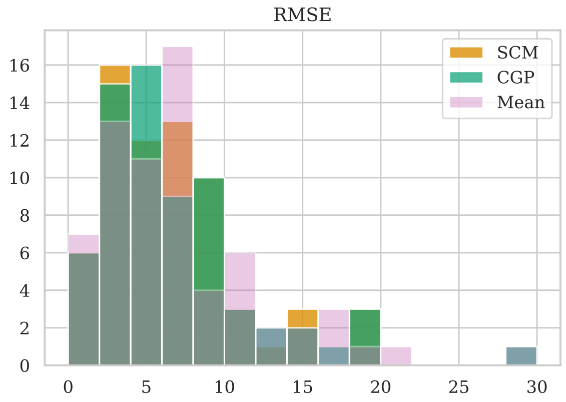

Supplementary Figure 1. Validating assumptions for Synthetic Controls Method (SCM) and conditional Gaussian Process (CGP) with placebo analysis: (left) we show the histogram for the root mean squared error (RMSE) between the observed data and predictions for the three methods - SCM, CGP and Mean estimate when performing the placebo analysis for different regions and control years. For CGP, we also show the corresponding 1-σ (68% confidence interval (c.l.)) estimated from the variance of the Gaussian model. We find that both CGP and SCM reduce RMSE as compared to SCM, providing better prediction.

(i.e. previous years) that reconstructs the potential outcome of the treated unit (current year) in the absence of pandemic.Then it estimates the counterfactual of the treated unit as this weighted combination of control units. The weights for various control units are estimated by minimizing the difference between the counterfactual and the observed data for the pre-pandemic period. In other words, we look for the set of weights (one weight per control year) which, when used to obtain the weighted average of 2014-2019 mortality time-series during pre-pandemic periods, most closely matches the pattern of mortality counts of the 2020 year. Thus if W is the weight vector for the control unit, then we minimize

$$W^* = \min_W (W^T \cdot X_0 - X_1^P)^2 \quad \text{s.t.} \sum_{i=i}^{N} W_i = 1, W_i > 0 \; \forall i \tag{4}$$

We have assumed a Gaussian, feature independent noise for the pre-pandemic data and put a positivity and unit L_1 norm constraint on the weights. Given these weights, the counterfactual is predicted as

$$Y_1 = W^* \cdot Y_0. \tag{5}$$

This prediction makes an implicit assumption that in the case of the counterfactual, the similarities between the years which lead to a good fit in weeks before the pandemic continue to exist in the later weeks as well and hence the same weights are used for the prediction It is a conservative assumption since there is no a-priori reason for this to not be the case for natural deaths in the absence of external interventions.

Placebo analysis : SCM and CGP, both the models make certain assumptions about the causal structure and the distribution of the data respectively. One way to validate these assumptions is with a placebo analysis. In this analysis, we in turn, treat every control unit year as a placebo treated unit and predict the mortality for this year by using the remaining control units and the data from this year before T_0, as we strive to do for 2020 in the main analysis. However since this placebo treated unit has not undergone any pandemic and has only seen natural deaths, the model predictions should ideally exactly match the observed data after T_0. We quantify any discrepancy in the observed and predicted data by estimating root mean squared error (RMSE) for each placebo example. We repeat this analysis for all three methods - SCM, CGP and historical mean for every control unit and every region in Italy. The histogram for all RMSE is shown in Supplementary Figure 1 and we find that overall, RMSE for CGP and SCM is smaller than a simple mean estimate, hence providing better prediction. This validates that our assumptions for SCM and CGP methods are well-founded and exploiting extra information from the pre-pandemic period helps our analysis.

1.1 Historical mortality and counterfactuals for different age groups

In this supplement, we show the total reported mortality and our counterfactual for different age groups in different regions to validate our baseline fits and support our estimates of excess deaths. Interestingly, we also note that while we see a decrease in

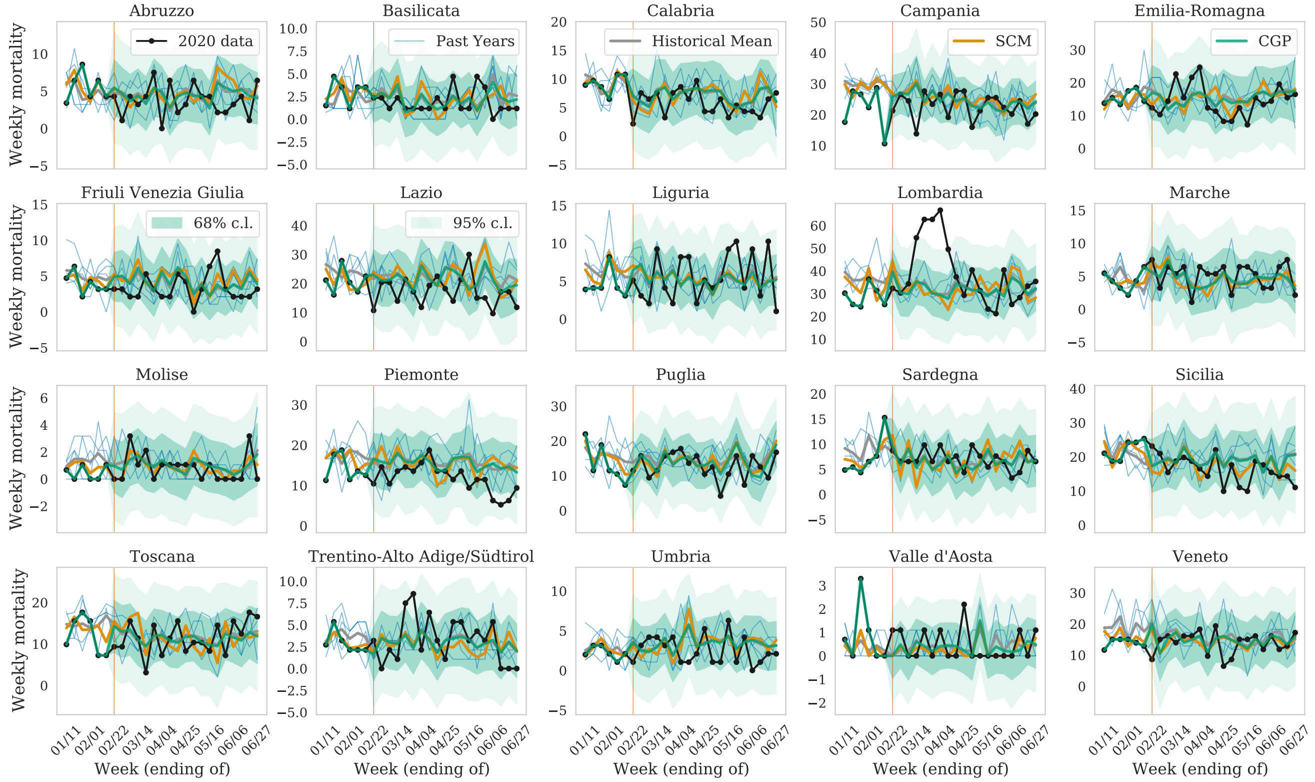

Supplementary Figure 2. Validating counterfactuals for the pre-pandemic data for age group 40 -49: we show the observed weekly mortality due to all causes for the period of January 1 - June 27 (black) in all 20 regions in Italy, and our prediction for the expected mortality in the absence of COVID-19 (conditional Gaussian Process (CGP) with its 1 and 2σ confidence interval in green and synthetic controls method (SCM) in orange). The first reported COVID-19 mortality occurred in the week ending on February 22 2020 (thin red vertical line). The historical data from 2015-2019 (blue) and corresponding historical mean (gray) is shown for comparison and is not a good fit to the observed pre-pandemic data. In dashed-black line, we also show the observed mortality after removing reported COVID-19 deaths.

the excess deaths over historical trends in the later months of the pandemic, we do not see a decrease in the total deaths for any age group.

1.2 Time-lag correlation analysis

We quantitatively assess the relation between measured excess deaths and official COVID-19 deaths by means of a two-parameter fit

$$\hat{y} = Ax(t - t'). \tag{6}$$

We allow the number of official COVID-19 fatalities, x, to be rescaled by a scalar amplitude A and shifted by a time offset t' to best match the observed excess deaths from our analysis, y. This fit is illustrated for Lombardia in Figure 6. We allow for an offset $t' \in [-15, 15]$, which limits the time window in which the fit is performed (yellow line). Animations of the fitting procedure are provided in the supplemental material.

we find that a negative time offset, meaning that the official death count trails the excess deaths, is clearly favored for all regions considered (Lombardia, Piemonte, Marche and Emilia-Romagna). This is illustrated in Figure 7, where we plot χ^2-values for each offset and region.

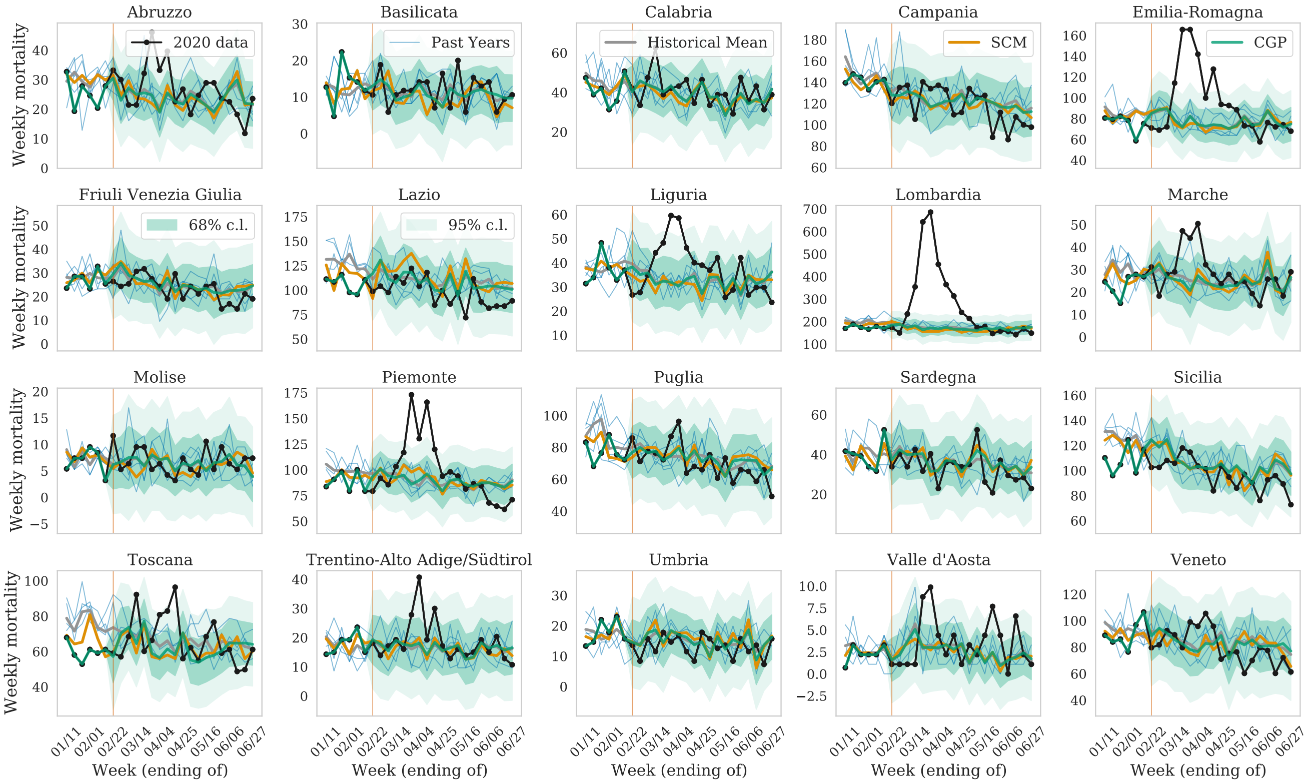

Supplementary Figure 3. Validating counterfactuals for the pre-pandemic data for age group 60-69: same as Supplementary Figure 2 but for age group 60-69.

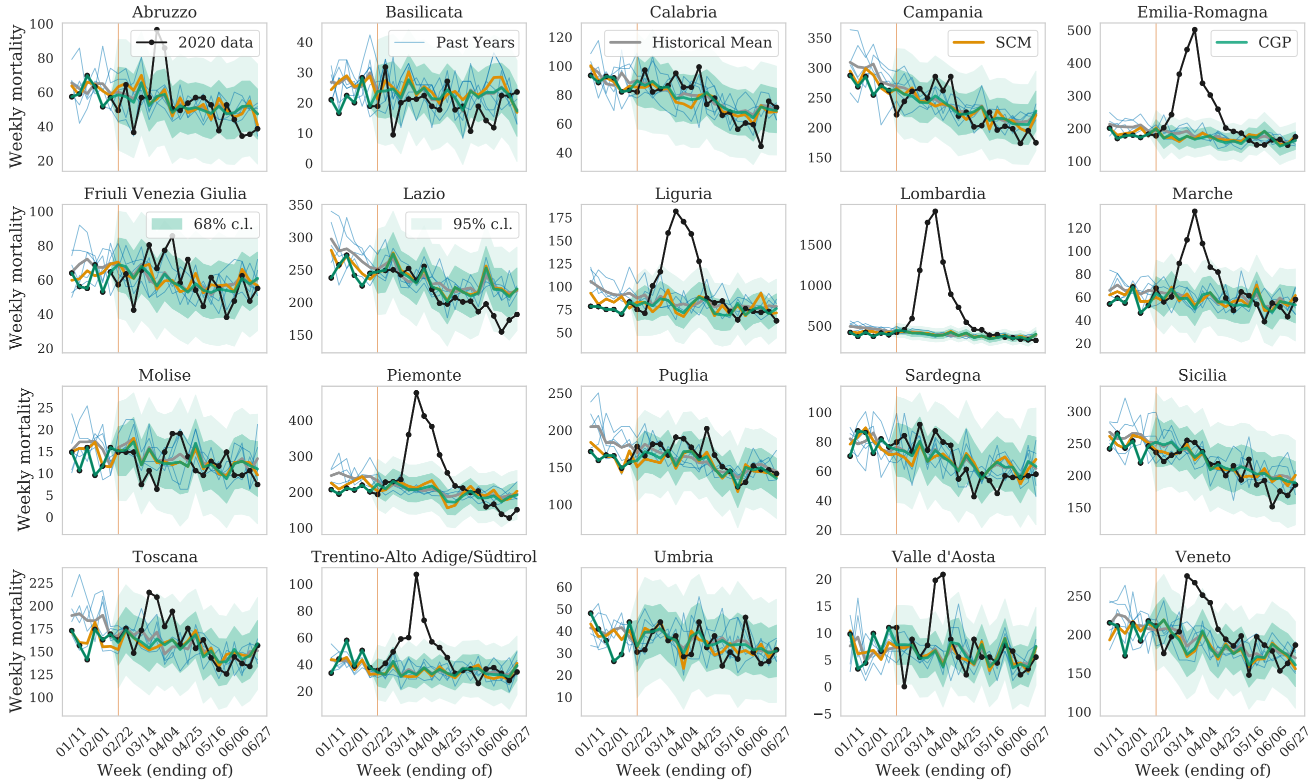

Supplementary Figure 4. Validating counterfactuals for the pre-pandemic data for age group 70-79: same as Supplementary Figure 2 but for age group 70-79.

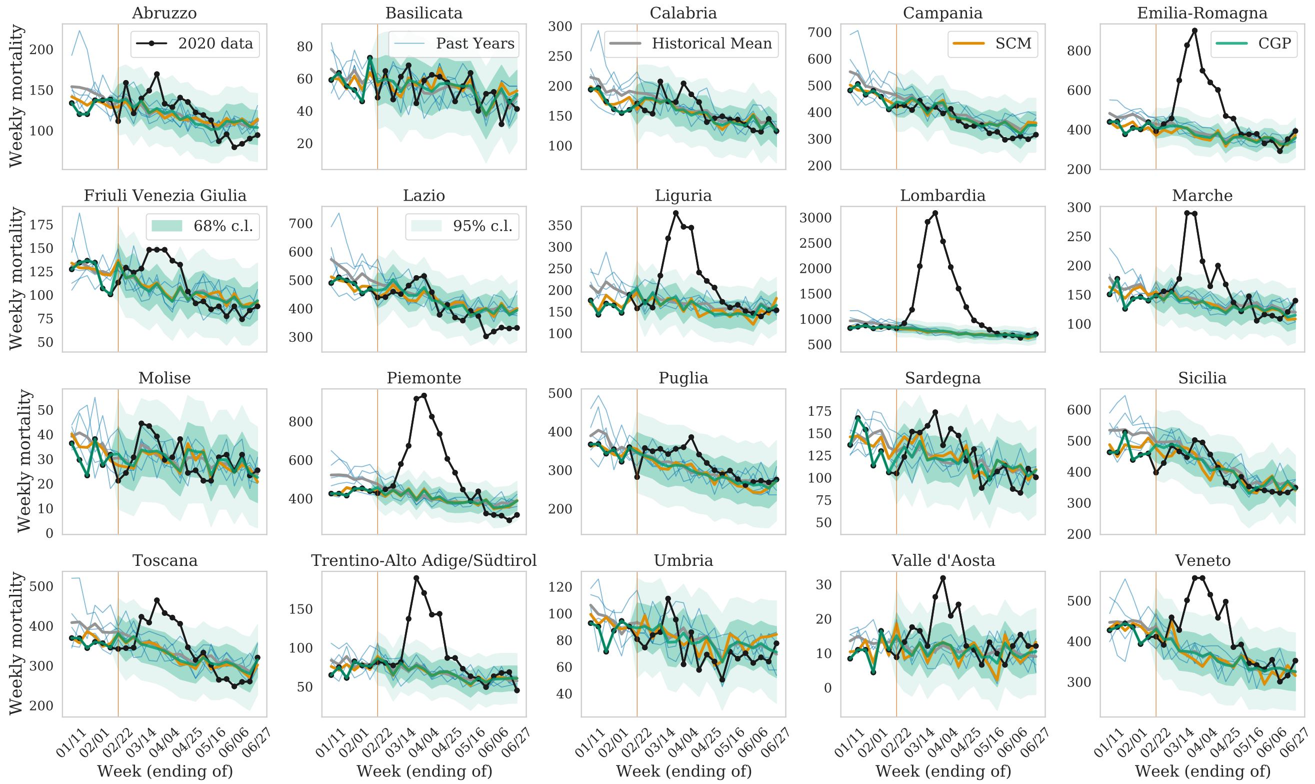

Supplementary Figure 5. Validating counterfactuals for the pre-pandemic data for age group 80-89: same as Supplementary Figure 2 but for age group 80-89.

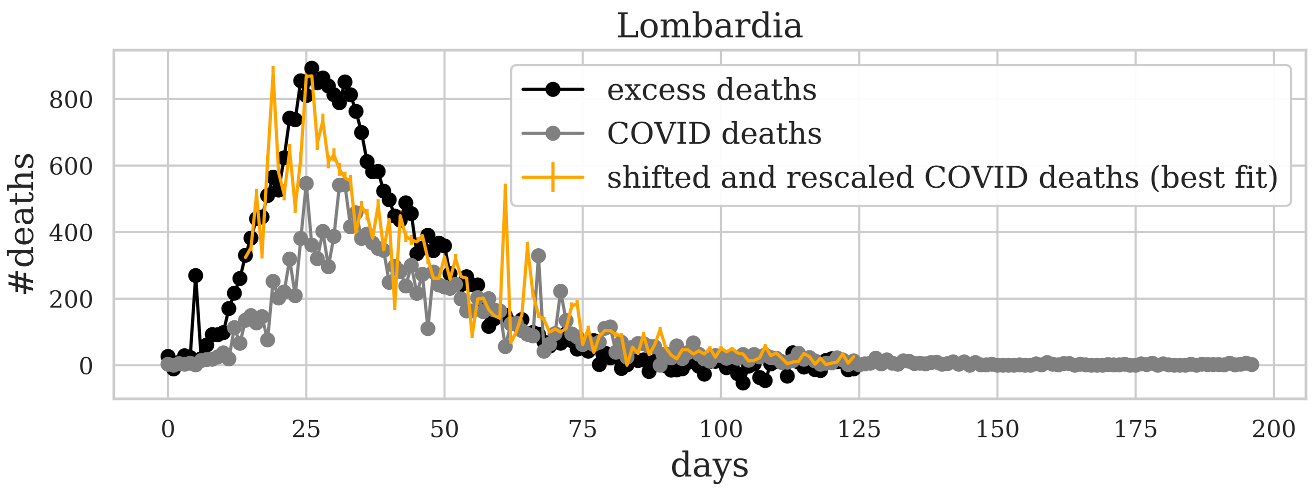

Supplementary Figure 6. Matching official COVID-19 deaths with measured excess deaths by means of a 2-parameter fit. In black we show the daily measured excess deaths, in gray the official COVID-19 death count. We allow the official COVID-19 death count to be rescaled and shifted (within a certain time window) to best match the excess death. The thus obtained best fit is shown in yellow.

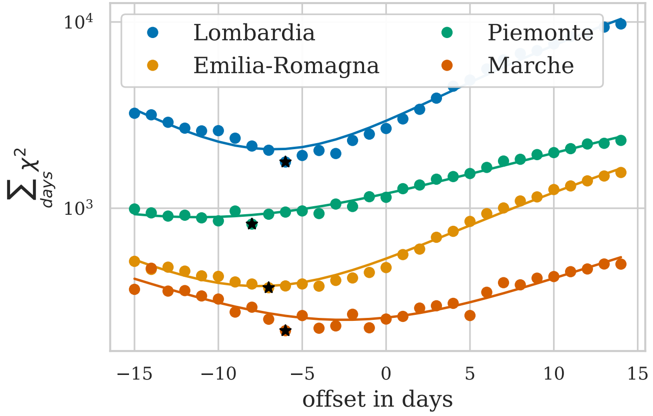

Supplementary Figure 7. 2-parameter fit χ^2's as a function of time-lag. We assess the confidence in our fits by plotting the χ^2 value for each fit. The resulting curves are well approximated by a quadratic curve (solid lines). The data from all regions clearly favor a negative offset. Lombardia, the region with the most COVID-19 cases, displays a well defined minimum, the time lags in other regions are associated with a larger uncertainty (wider minima).

This analysis establishes a correlation between excess deaths and official death counts, but not necessarily causality. However, it is a strong indication that the two have the same origin. We can only speculate about the reason for the observed time-lag. It could be that official numbers mostly account for deaths that occur in hospitals, where they occur delayed because of better treatment, but it could also reflect changes in testing strategies an availabilities with time.

Supplementary References

1. Modi, C. & Seljak, U. Generative Learning of Counterfactual for Synthetic Control Applications in Econometrics. *arXiv e-prints* arXiv:1910.07178 (2019). 1910.07178.
2. Abadie, A., Diamond, A. & Hainmueller, J. Synthetic control methods for comparative case studies: Estimating the effect of california's tobacco control program. *J. Am. Stat. Assoc.* **105**, 493–505, DOI: 10.1198/jasa.2009.ap08746 (2010).
